# Treatment with Thyme Essential Oil Delays Loss Reductions in Postharvest Chinese Flowering Cabbage

**DOI:** 10.3390/foods14213704

**Published:** 2025-10-29

**Authors:** Daoye Chen, Minhui Li, Wenya Wu, Ling Wang, Yulong Chen, Fuwang Wu

**Affiliations:** 1 School of Food Science and Engineering, Foshan University, Foshan 528225, China; 18898609320@163.com (D.C.); 18312092150@163.com (M.L.); 15918088320@163.com (W.W.); 2 Sericultural & Agri-Food Research Institute Guangdong Academy of Agricultural Sciences, Key Laboratory of Functional Foods, Ministry of Agriculture and Rural Aﬀairs, Guangdong Key Laboratory of Agricultural Products Processing, Guangzhou 510610, China; wangl12l@163.com (L.W.); chenyulong@gdaas.cn (Y.C.); 3 Guangdong Provincial Key Laboratory of Intelligent Food Manufacturing, Foshan University, Foshan 528225, China

**Keywords:** Chinese flowering cabbage, thyme essential oil, yellowing, senescence, preservation

## Abstract

Leaf yellowing is the primary indicator of deterioration in the quality of Chinese flowering cabbage. In this study, Chinese flowering cabbage (*Brassica rapa* var. Parachinensis), a perishable, freshly cut vegetable, was treated with 0.5 ppm thyme essential oil and stored at 15 °C with 90% relative humidity (RH), with the aim of exploring its preservation effect. Compared with the control group, the treatment postponed the yellowing and chroma decline of leaves by 24% and 60%, respectively, inhibited the degradation of chlorophyll by 62% and the increase in relative conductivity by 62%, and sustained high contents of vitamin C (0.238 mg/g), soluble solids (8.5%), soluble sugar (2.658 mg/g), and soluble protein (5.294 mg/g) after 7 days of storage. Moreover, the rising activity of peroxidase (POD) during treatment was slowed during storage, alleviating lignification, while the activity of catalase (CAT) was dramatically enhanced in the late storage stage, avoiding oxidative damage and improving storage quality. Overall, thyme essential oil treatment significantly delayed the senescence of Chinese flowering cabbage and demonstrated considerable potential as a natural preservative.

## 1. Introduction

Chinese flowering cabbage is a commonly cultivated vegetable crop in South China and is largely exported to Southeast Asian, European, and American countries due to its abundant nutrients, unique flavor, and crisp taste [1]. However, like most green leafy vegetables, Chinese flowering cabbage still undergoes vigorous respiratory metabolism after harvest and easily suffers from appearance quality deterioration during storage, which mainly manifests as etiolated leaves, chlorophyll content reduction, and increases in cell membrane permeability due to the high specific surface area and water content. It is prone to corruption during storage and transportation due to fungal and bacterial infections such as downy mildew, soft rot, anthracnose, black rot, and black spots. Moreover, improper storage temperature, relative humidity, or packing can accelerate deterioration, resulting in huge economic losses. At present, the methods applied in vegetable storage mainly include low-temperature, chemical, and modified atmosphere preservation.

Studies have shown that cold storage combined with controlled atmosphere (CA) treatment is highly effective in preventing Chinese flowering cabbage stalks from losing turgor and delaying leaf yellowing [2]. In addition, exogenous application of N-phenyl-N-(2-chloro-4-pyridyl) urea (CPPU) treatment significantly delayed senescence, reduced ROS accumulation, helped to maintain membrane integrity and energy status, and delayed rapid chlorophyll degradation, which is beneficial for ensuring the nutritional quality of Chinese flowering cabbage [3]. However, low-temperature refrigeration may cause cold injury and requires high energy consumption, chemical preservation methods may result in food safety and environmental pollution problems, and CA storage may be affected by fluctuations in gas composition, as well as entailing high equipment costs. These factors limit such methods’ application and extension. With improving life quality, developing safe, green, and efficient preservation methods has become an urgent requirement for fruit and vegetable storage.

Plant essential oils are recognized as generally considered safe (GRAS), and they not only provide nutrition for humans but also have natural antiseptic effects [4]. As an important source of natural preservatives, plant essential oils form a bacteriostatic atmosphere around foods through direct contact or fumigation [5], and there have been numerous reports of their preservation effect on fruits. Studies show that thyme oil can suppress the growth of *Colletotrichum gloeosporioides* and *Rhizopus stolonifer*, thus improving the microbial resistance of papaya fruit and prolonging its shelf life [6]. Peppermint oil can restrain mold growth, thus exerting an antibacterial effect and extending the storage time of postharvest dragon fruit [7]. In addition, plant essential oils have good fresh-keeping effects on fruits such as citrus [8], tomatoes [9], Jujube [10], and apples [11], and they can inhibit the occurrence of decay, prevent quality deterioration, and extend the fruit storage process. However, few reports have focused on the effect of plant essential oils on delaying the senescence of leaf vegetables.

Chlorophyll content is an important indicator of vegetable storage quality. Results have shown that 1-methylcyclopropene (1-MCP) treatment can effectively alleviate chlorophyll degradation in broccoli after harvest, helping to maintain its green color [12]. In addition, exogenous sodium nitroprusside can also delay the degradation of chlorophyll, thereby prolonging the shelf life of broccoli [13]. Furthermore, melatonin treatment not only exerts a certain retardation effect on the degradation of chlorophyll in Chinese flowering cabbage leaves but also suppresses the expression of senescence-associated genes [14]. Moreover, by restricting the expression of abscisic acid (ABA) synthesis-related genes, it can postpone leaf senescence [15]. Interestingly, up-regulated expression of melatonin synthesis-related genes increased internal source melatonin content, while down-regulated expression of ABA synthesis-related genes decreased ABA content, which ultimately retarded leaf senescence in cabbage [16]. Our previous research showed that treatment with 15 ppm peppermint essential oil delayed banana ripening and inhibited the degradation of chlorophyll in the peel [17]. Moreover, 5 mg L^−1^ ginger essential oil significantly slowed the degradation of both chlorophyll a and b in the fern [18]. Therefore, the physiological function of plant essential oils in delaying vegetable quality deterioration and chlorophyll degradation needs to be further elucidated.

Thyme essential oil is a rich aromatic volatile substance extracted from thyme (*Thymus vulgaris* L.) that presents great antibacterial and antioxidant effects and has been widely applied in the pharmaceutical chemical engineering and food industry [19]. A previous study suggested that thyme oil could potentially be used for controlling fusariosis in postharvest pineapples [20]. Moreover, thyme essential oil evaporation combined with controlled atmosphere treatment could significantly maintain the appearance of strawberries with a high commercial value [21]. Inspired by these studies, three plant essential oils or plant essential oil extracts, including thyme essential oil, eugenol, and carvacrol, were applied to treat Chinese flowering cabbage in our preliminary experiment, and thyme essential oil was found to be the most effective for retarding leaf etiolation (Figures S1 and S2). Therefore, thyme essential oil may not only contribute anticorrosion and preservation effects but may also be involved in delaying the aging process of Chinese flowering cabbage.

While the efficacy of plant essential oils as natural preservatives for postharvest fruits is well established, their application in the preservation of leafy vegetables remains relatively unexplored. This gap is particularly pronounced for highly perishable commodities like Chinese flowering cabbage, which suffers from rapid postharvest senescence and quality deterioration. In this study, Chinese flowering cabbage was treated with thyme essential oil to explore its preservation effect on the appearance and nutritional quality of the crop during storage. We also examined the antioxidant enzyme activities of the oil. We aimed to gain a deeper understanding of the preservation mechanism of thyme essential oil used to treat Chinese flowering cabbage, extending beyond its known functions and providing novel, targeted insights into the use of plant essential oils. Finally, this study serves as a theoretical foundation for the application of thyme essential oil in retarding vegetable senescence.

## 2. Materials and Methods

### 2.1. Materials and Treatments

Chinese flowering cabbage (*Brassica rapa* var. Parachinensis) was purchased from Zhongnan agricultural products wholesale market (Guangdong Province, China), and specific crops were chosen for their uniform size, maturity, and lack of pests or mechanical damage. In our previous pilot experiment, equivalent materials (weighing 750 g per treatment) were placed into a storage box (24 L), followed by adding different concentrations (0, 12, and 24 μL, which made the final concentration 0, 0.5, and 1 ppm, respectively) of thyme essential oil (S26577, purchased from Shanghai Yuanye Bio-Technology Co., Ltd., Shanghai, China) to a piece of filter paper inside the box for sustained release. The box was immediately sealed with a cap and stored at 15 °C with 90% RH for observation and photographing (Figure S1). Finally, 0.5 ppm thyme essential oil treatment was selected for the repeated experiment. On days 0, 1, 3, 5, and 7 of storage, samples from three biological replicates were used for the measurement of yellowing rate, color difference, electrical conductivity, and total soluble solids. Meanwhile, the leaf tissue was sampled, immediately frozen in liquid nitrogen, and stored at −80 °C for further analysis of the contents of chlorophyll, vitamin C, soluble sugar and soluble protein, and the activities of antioxidant enzymes.

### 2.2. Measurement of Yellowing Rate

According to the measurement of Fang et al. [22], the yellowing area of Chinese flowering cabbage leaves was classified into 5 levels (grades 0, 1, 2, 3, and 4 account for 0%, 0–25%, 25–50%, 50–75%, and 75–100% of the yellowing area, respectively), which were used to calculate the yellowing index as follows: yellowing index (%) = Σ (yellowing grade × leaf number of this grade)/(total number of leaves × representative value of highest grade) × 100%.

### 2.3. Color Difference Assay

The Delta E of leaves was detected by using a color difference meter (CR-10, Konica Minolta, Japan). A total of 30 locations were determined for each treatment to record the lightness L values, saturation C values, and chroma H values. The average and standard deviation were calculated.

### 2.4. Determination of Chlorophyll Content

Chlorophyll content was determined based on the method of Li et al. [3]. An extracting solvent was prepared by mixing acetone and absolute ethanol at a volume ratio of 2:1 and kept away from light before operation. Chinese flowering cabbage tissue was ground with liquid nitrogen, and 1 g of the powder was weighed and placed in a centrifuge tube, followed by adding 0.05 g of CaCO_3_ powder and 5 mL of the extracting solvent. The sample was filtered into a brown volumetric flask, and the final volume was adjusted to 25 mL with the solvent. Then, the absorbance of the extract was measured at 645 and 663 nm and used for calculating the content of chlorophyll (CT) through the following formulas: C_a_ (mg/L) = 12.7 × OD_663nm_ − 2.69 × OD_645nm_, C_b_ (mg/L) = 22.9 × OD_645nm_ − 4.68 × OD_663nm_, and CT (mg/L) = C_a_ + C_b_ = 8.02 × OD_663nm_ − 20.21 × OD_645nm_.

### 2.5. Determination of Relative Electrical Conductivity

Relative electrical conductivity was measured according to the method of Xu et al. [23]. Circular tissues with a diameter of 0.5 cm were cut from the treatment leaves by using a hole punch, and then ten discs were put into a conical flask with 25 mL distilled water. After 30 min of gentle shaking at room temperature, the solution was measured using a conductivity meter to calculate the C1 value and weighed before immersion in a boiling water bath for 5 min. Afterward, the electrical conductivity C2 value was determined by supplementing with distilled water to maintain a constant weight and allowing it to cool down to room temperature. The assay was repeated three times, and the average value was taken. The formula for calculating the relative electrical conductivity of the leaf is as follows: relative electrical conductivity (%) = C1/C2 × 100%.

### 2.6. Vitamin C Content Analysis

Vitamin C content was assessed according to the method of Lo’ay and El-Khateeb [24] with a slight modification. One gram of Chinese flowering cabbage tissue powder, ground with liquid nitrogen, was weighed and placed into a centrifuge tube and then diluted with an oxalic acid solution (20 g/L) to 25 mL and kept at 4 °C for 10 min for extraction with gentle shaking. After centrifugation at 10,000× *g* and 4 °C for 15 min, a 10 mL filtrate was titrated against a freshly prepared 2, 6-dichlorophenol indophenol volumetric solution, and consumption (V1) was recorded until the sample maintained a pink color for 15 s. Simultaneously, a 10 mL oxalic acid solution (20 g/L) was also titrated against the volumetric solution and set as the blank (V_0_). The following formular was used for calculation: Ascorbic acid content of Chinese flowering cabbage (mg/g) = Total extraction volume (V) × (Consumption volume of sample V_1_—Consumption volume of blank V_0_) × Equivalent ascorbic acid mass of 1 mL 2, 6-dichloroindophenol indophenol solution (ρ)/(Titrated volume V_s_ × Weight of leaf tissues m). The above experiments were conducted three times in parallel.

### 2.7. Determination of Soluble Solids Content

Three grams of leaf tissue were weighed and ground in a mortar to obtain the filtrate through 8 layers of gauze. The filtrate was measured by using a saccharimeter (VBR20) to determine the content of soluble solids. Distilled water was set as the blank. The experiment was repeated three times, and the average value was recorded.

### 2.8. Determination of Soluble Sugar Content

Soluble sugar content was measured according to the anthrone colorimetric method [25]. One gram of Chinese flowering cabbage tissue powder that had been ground with liquid nitrogen was weighed and placed into a centrifuge tube, followed by adding 15 mL distilled water and heating in a water bath (80 °C) for 30 min. After centrifugation at 4000× *g* for 10 min at room temperature, the supernatant was diluted to 100 mL with distilled water. Then, 1 mL of the final diluent and 5 mL of concentrated sulfuric acid–anthrone reagent were mixed and heated for 10 min in boiling water. After cooling quickly to room temperature, the absorbance of the samples was measured and recorded at a wavelength of 620 nm, performing three analyses for parallel determination. Finally, the soluble sugar content was calculated according to the standard curves of glucose.

### 2.9. Determination of Soluble Protein Content

Soluble protein was measured following the method of Huang et al. [2] with some modifications. One gram of Chinese flowering cabbage tissue powder, ground with liquid nitrogen, was placed in a centrifuge tube and suspended in 15 mL of distilled water. After centrifugation at 4000× *g* for 10 min at room temperature, the supernatant was diluted to 50 mL with distilled water. Then, a 1 mL sample solution was mixed with 4 mL Bradford working reagent, and the absorbance of the reaction solution was recorded at a wavelength of 595 nm after standing for 5 min. The soluble protein content was finally calculated according to the standard curve of bovine serum albumin, and the measurement was repeated three times in parallel.

### 2.10. Assay for POD Activity

POD activity was measured using the method described by Zhang et al. [26] with slight amendments. Two grams of Chinese flowering cabbage tissue powder, ground with liquid nitrogen, were placed into a centrifuge tube, followed by adding 10 mL of pre-cooled PBS solution (0.05 mol/L, containing 4% PVP, pH = 7.0), and extracted in an ice bath for 20 min. After centrifugation at 80,000× *g* at 4 °C for 20 min, the supernatant obtained was a crude enzyme. The measuring system of POD activity contained 0.1 mL 0.46% H_2_O_2_, 0.1 mL 4% supersaturated guaiacol, and 2.75 mL PBS solution (0.05 mol/L, pH = 7.0), followed by mixing 0.05 mL crude enzyme to start the reaction. The change in absorbance at 470 nm was recorded every 15 s for 3 min. One unit of activity was defined as the amount of enzyme that changed the absorbance by 0.001 at 470 nm within 1 min. All measurements were repeated three times.

### 2.11. Measurement of CAT Activity

CAT activity was determined according to the method of Zhang et al. [26] with modifications. The CAT crude enzyme was prepared in the same way as POD. Enzyme activity was measured by adding 3 mL 0.01 mol/L H_2_O_2_ and putting it into a quartz colorimetric instrument, followed by mixing 0.1 mL enzyme solution to start the reaction. The absorbance value was recorded immediately every 15 s for 2 min at a wavelength of 240 nm, and one unit of activity was defined as the amount of enzyme that varied the absorbance by 0.001 within 1 min. The PBS (0.05 mol/L, pH = 7.0) solution was set as the blank reference, and the measurement was repeated three times in parallel.

### 2.12. Data Analysis

All experiments described were performed in technical triplicate. Excel 2010 was used for data statistics, SigmaPlot 10.0 was applied for chart drawing, and SPSS 25.0 (paired-sample *t*-test) was adopted for significance analysis. * Indicates a significant difference (*p* < 0.05) between the control group and the 0.5 ppm thyme essential oil treatment group under the same storage time; ** indicates an extremely significant difference (*p* < 0.01) between the control group and the 0.5 ppm thyme essential oil treatment group under the same storage time.

## 3. Results and Discussion

### 3.1. Thyme Essential Oil at 0.5 ppm Can Delay the Deterioration in the Appearance of Chinese Flowering Cabbage During Storage

**Figure 1:**
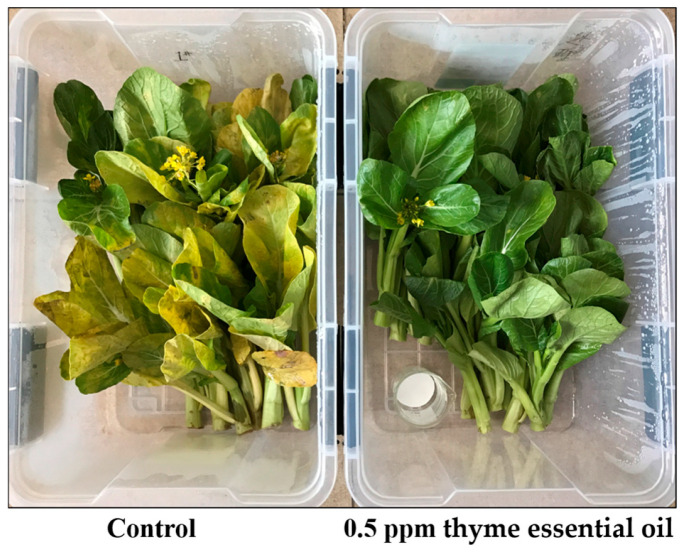
Effect of 0.5 ppm thyme essential oil treatment on appearance of Chinese flowering cabbage after storage at 15 °C for 6 days.

In this study, after storage for 6 days under conditions of 15 °C and 90% RH, Chinese flowering cabbage treated with 0.5 ppm thyme essential oil was kept fresh and edible, while the control experienced leaf etiolation, flowering, and even decay and completely lost its commodity value (Figure 1).

**Figure 2:**
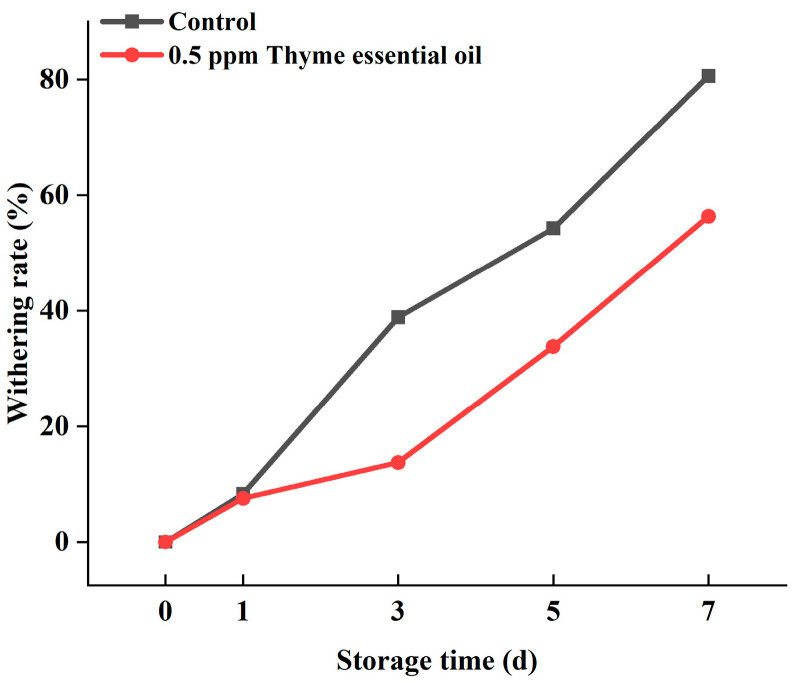
Effect of 0.5 ppm thyme essential oil treatment on withering rate of Chinese flowering cabbage during storage for 7 days.

Yellowing was considered the main reason for appearance quality deterioration in Chinese flowering cabbage during storage, the commodity rate of which can be judged by measuring the yellowing index. In this study, the withering rate of Chinese flowering cabbage leaves increased rapidly, and the cabbage finally lost its commercial value after 7 days. However, the yellowing index of the 0.5 ppm thyme essential oil treatment group was significantly lower than that of the control during storage, showing that this treatment effectively delayed Chinese flowering cabbage yellowing and protected the appearance of the cabbage (Figure 2).

**Figure 3:**
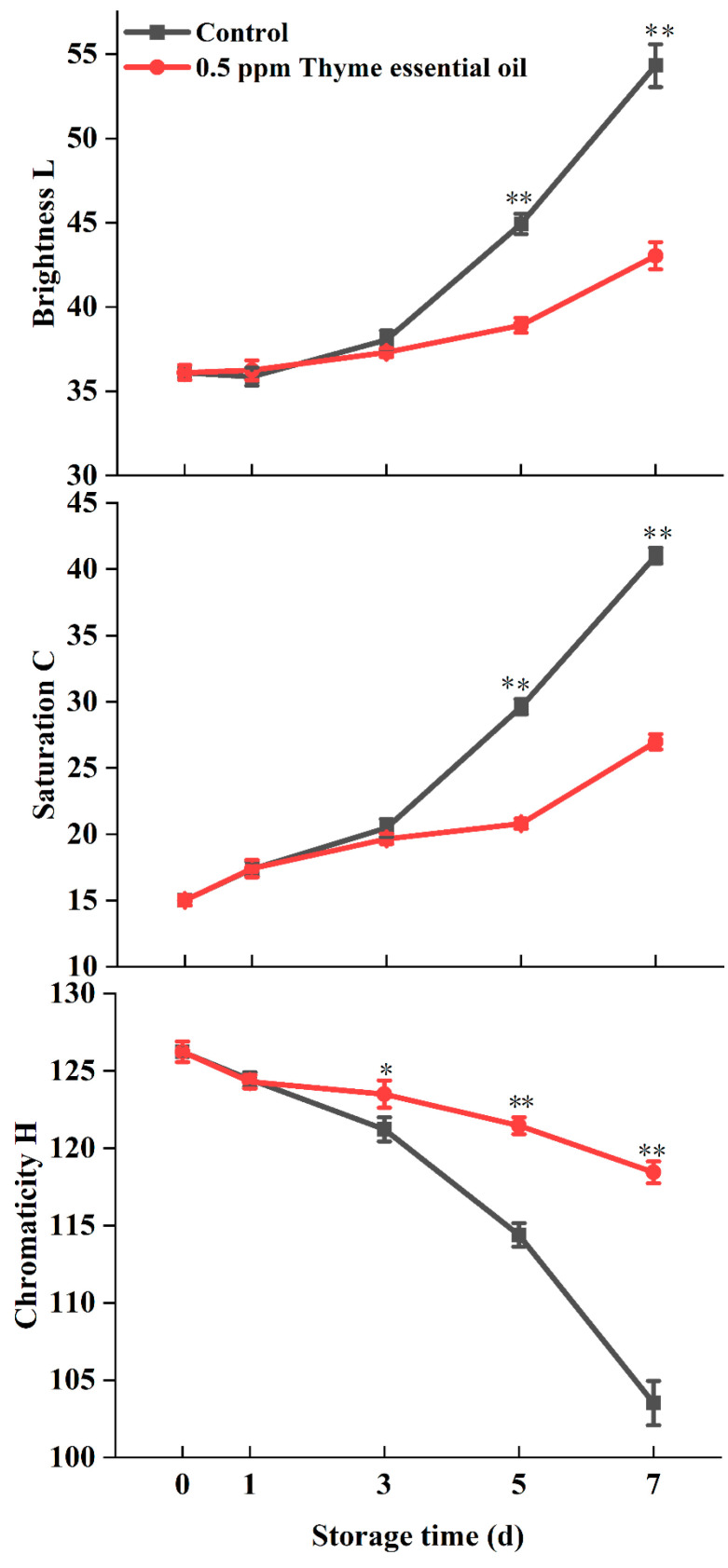
Effect of 0.5 ppm thyme essential oil treatment on the color difference (L, C, H value) of Chinese flowering cabbage during storage for 7 days. Each value represents the means ± SE of three replicates. Asterisks indicate significant differences (* *p* < 0.05, ** *p* < 0.01) between the 0.5 ppm thyme essential oil treatment group and the control at each time point during storage.

Measuring the color difference is a common method for evaluating the surface quality of vegetables. The results showed that the brightness and saturation values of Chinese flowering cabbage leaves continuously increased with storage time, while the chroma value decreased gradually, exhibiting a consistent variation trend with the yellowing of leaves (Figure 3). Meanwhile, the 0.5 ppm thyme essential oil treatment group showed a delay in the increase in brightness and saturation values and in the decrease in the chroma value. Moreover, there were significant differences compared with the control group. Therefore, it can be said that 0.5 ppm thyme essential oil treatment can delay the change in the color difference in Chinese flowering cabbage leaves.

**Figure 4:**
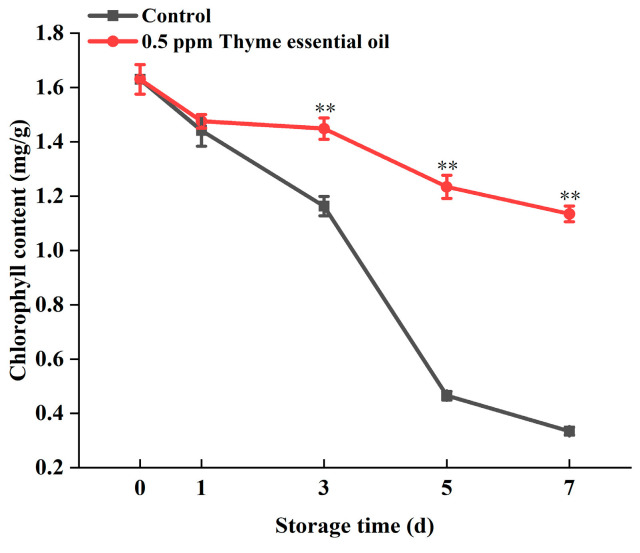
Effect of 0.5 ppm thyme essential oil treatment on the content of chlorophyll in Chinese flowering cabbage during storage for 7 days. Each value represents the means ± SE of three replicates. Asterisks indicate significant differences (** *p* < 0.01) between the 0.5 ppm thyme essential oil treatment group and the control at each time point during storage.

In addition, the chlorophyll content, which determines the green color of Chinese flowering cabbage and directly affects its freshness, gradually degrades with maturation and senescence in plants [27]. Although exogenous melatonin and cytokinin treatments could inhibit the decomposition of chlorophyll in cabbage, the application of plant hormone preservatives still has limitations [28]. The chlorophyll content of the control samples decreased sharply during storage for 7 days, dropping to less than 25% of the content observed one day after the harvest. However, chlorophyll degradation in the 0.5 ppm thyme essential oil treatment group proceeded relatively slowly, and about 75% of the chlorophyll content was preserved after storage for 7 days (Figure 4). Therefore, 0.5 ppm thyme essential oil treatment effectively inhibits the degradation of chlorophyll in Chinese flowering cabbage leaves.

**Figure 5:**
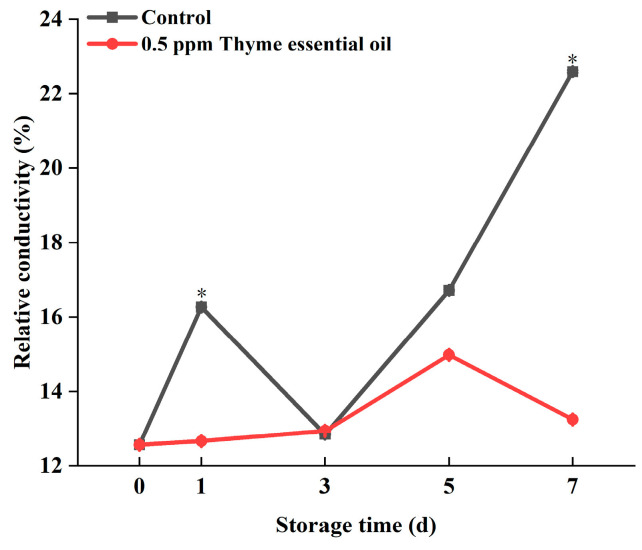
Effect of 0.5 ppm thyme essential oil treatment on the relative conductivity of Chinese flowering cabbage during storage for 7 days. Each value represents the means ± SE of three replicates. Asterisks indicate significant differences (* *p* < 0.05) between the 0.5 ppm thyme essential oil treatment group and the control at each time point during storage.

In the process of plant ripening and senescence, cell membranes can be oxidized by a variety of harmful substances, like reactive oxygen species, which leads to a constantly rising relative conductivity. Therefore, the relative conductivity can reflect the aging degree of fruits and vegetables to some extent. In this experiment, the relative conductivity of Chinese flowering cabbage in the control group was dramatically increased with prolonged storage time, while that of the 0.5 ppm thyme essential oil treatment group increased slowly and maintained a constant low level (Figure 5). Consequently, 0.5 ppm thyme essential oil treatment delays the senescence of Chinese flowering cabbage and protects cell integrity.

Zhang et al. [28] showed that the biodegradation process of chlorophyll is associated with the expression of PPH (pheophytin and pheophorbide hydrolase gene), PAO (pheophorbide a and oxygenase gene), and RCCR (red chlorophyll degradation product reductase gene). In particular, the activities of chlorophyllase and magnesium removal chelatase played a key role, and ethylene treatment could accelerate the aging process of Chinese flowering cabbage. Fan et al. [29] found that the WRKY65 transcription factor could participate in the degradation of chlorophyll by regulating the expression of aging-related genes such as BrNYC1 (non-yellow coloring1/Chl b reductase), BrSGR1 (stay-green1), and BrDIN1 (dark-inducible). The NAC055 transcription factor is an activator of transcription in the production of reactive oxygen species (ROS) and chlorophyll degradation, and is also involved in the senescence process of Chinese flowering cabbage [30]. Furthermore, the ERF72 transcription factor of the ethylene signal transduction pathway not only induces the expression of SAGs (senescence-associated genes) and CCGs (chlorophyll metabolism genes), but also activates the expression of key enzyme genes (LOX4, AOC3, and OPR3) of jasmonic acid biosynthesis, indirectly participating in the aging process of Chinese flowering cabbage leaves [31]. Therefore, it was hypothesized that 0.5 ppm thyme essential oil treatment might inhibit the expression of transcription factors, including WRKY65, NAC055, and ERF72, and reduce the transcription of aging-related genes of chlorophyll degradation and ROS metabolism, thus delaying the postharvest senescence process and maintaining the appearance of Chinese flowering cabbage during storage.

### 3.2. Thyme Essential Oil Treatment at 0.5 ppm Can Maintain the Nutritional Quality of Flowering Chinese Cabbage During Storage

Chinese flowering cabbage is rich in nutrients, but they are easily lost during storage, such as vitamin C, soluble sugar, and soluble protein. Plant essential oils not only have anticorrosion, sterilization, and antioxidation effects but also help to preserve the nutritional quality of fruits and vegetables during storage. Studies have shown that thyme essential oil exhibits antimicrobial effects against Listeria in fresh-cut produce [32]. In addition, it can delay nutrient loss and play an effective role in extending the postharvest shelf life of jujube fruit when combined with rosemary and cinnamon essential oils [33]. Moreover, thyme essential oil combined with chitosan could also inhibit vitamin C loss in postharvest shiitake mushrooms and extend their shelf life [34].

**Figure 6:**
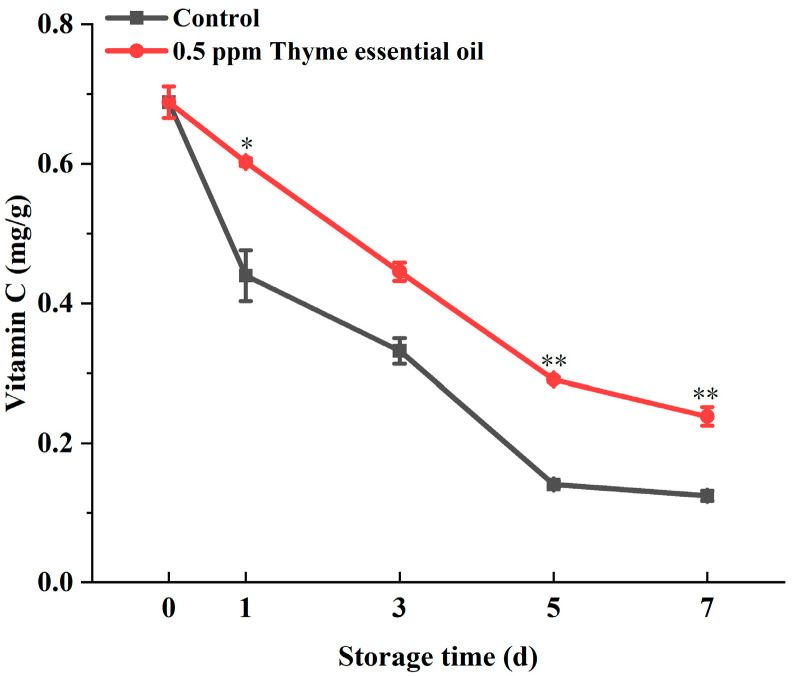
Effect of 0.5 ppm thyme essential oil treatment on vitamin C content in Chinese flowering cabbage during storage for 7 days. Each value represents the means ± SE of three replicates. Asterisks indicate significant differences (* *p* < 0.05, ** *p* < 0.01) between the 0.5 ppm thyme essential oil treatment group and the control at each time point during storage.

Vitamin C is one of the main nutrients in Chinese flowering cabbage. However, vitamin C easily degrades during the storage of fruits and vegetables. In our experiment, the vitamin C content of Chinese flowering cabbage in the control group decreased easily with storage time, while the vitamin C degradation rate of the 0.5 ppm thyme essential oil treatment group showed rapid progress and increased significantly during storage (Figure 6). Our studies showed that vitamin C can be oxidized by reacting with reactive oxygen species. In addition, enzymes such as peroxidase or ascorbate oxidase can also break down vitamin C. Moreover, the bioactive compounds of thyme essential oil can reduce the activity of oxidase and protect against free radicals, thus delaying the oxidation of vitamin C [35]. Therefore, 0.5 ppm thyme essential oil treatment can maintain the high content of vitamin C in Chinese flowering cabbage during storage.

**Figure 7:**
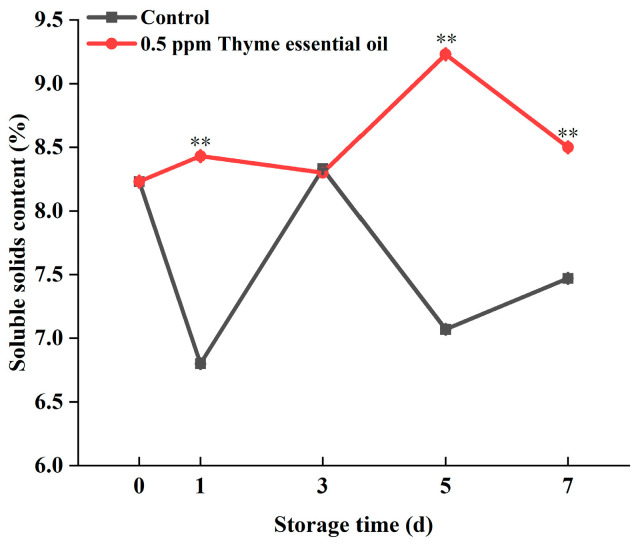
Effect of 0.5 ppm thyme essential oil treatment on soluble solids content in Chinese flowering cabbage during storage for 7 days. Each value represents the means ± SE of three replicates. Asterisks indicate significant differences (** *p* < 0.01) between the 0.5 ppm thyme essential oil treatment group and the control at each time point during storage.

The soluble solids content is another important index for evaluating the storage quality of fruits and vegetables, including sugars, organic acids, and minerals. It is closely related to the respiration rate and maturity of fruits and vegetables [36]. Thyme essential oil can reduce the respiration rate of fruits and vegetables, thereby slowing their metabolism, reducing the consumption of soluble solids, and delaying the ripening process [37]. In this study, the soluble solids content of the control group decreased after harvest, followed by a slight rise during storage, while the 0.5 ppm thyme essential oil treatment delayed the peak in soluble solids content, which remained at a higher level during the storage period (Figure 7). Thus, 0.5 ppm thyme essential oil treatment helped maintain the soluble solids of Chinese flowering cabbage.

**Figure 8:**
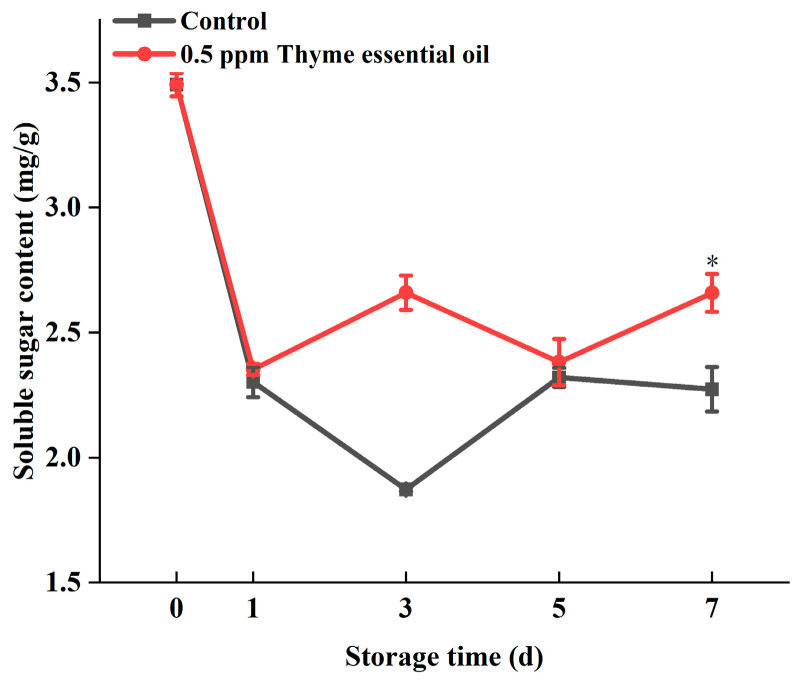
Effect of 0.5 ppm thyme essential oil treatment on soluble sugar content in Chinese flowering cabbage during storage for 7 days. Each value represents the means ± SE of three replicates. Asterisks indicate significant differences (* *p* < 0.05) between the 0.5 ppm thyme essential oil treatment group and the control at each time point during storage.

Soluble sugar is another major nutritional component in fruits and vegetables. The main feature of Chinese flowering cabbage is its sweetness, so its soluble sugar content is an important indicator for its quality assessment. With the increase in leaf yellowing during storage, the progress of aging was obvious, and the soluble sugar content decreased with the increase in storage time [38]. During storage, the soluble sugar content of Chinese flowering cabbage decreased sharply after harvest in the control group, and its content dropped to less than 60% of the preharvest value. In contrast, the 0.5 ppm thyme essential oil treatment group maintained a relatively high soluble sugar content (Figure 8). Therefore, this shows that 0.5 ppm thyme essential oil treatment can avoid the rapid decomposition of soluble sugar to some extent in Chinese flowering cabbage.

**Figure 9:**
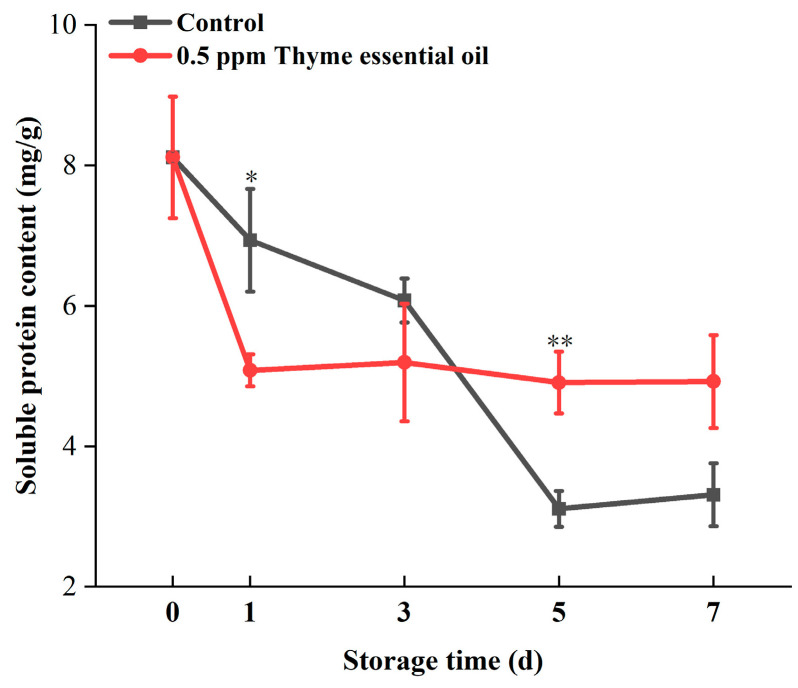
Effect of 0.5 ppm thyme essential oil treatment on soluble protein content in Chinese flowering cabbage during for 7 days. Each value represents the means ± SE of three replicates. Asterisks indicate significant differences (* *p* < 0.05, ** *p* < 0.01) between the 0.5 ppm thyme essential oil treatment group and the control at each time point during storage.

Leaf senescence accelerated the deterioration in the quality of cabbage, and soluble protein decreased with the increase in storage time [2]. In this study, the soluble protein content of Chinese flowering cabbage decreased rapidly during the storage period, but the 0.5 ppm thyme essential oil treatment group exhibited a relatively stable soluble protein content during preservation, especially during anaphase storage, where the soluble protein content was higher than the control (Figure 9). The measurement results indicate that 0.5 ppm thyme essential oil treatment can delay the degradation of soluble protein in Chinese flowering cabbage.

The nutritional quality of Chinese flowering cabbage after harvest is mostly affected by respiratory metabolism, especially after mechanical damage, which can promote the production of ethylene, enhance the respiratory rate, and accelerate aging [38]. Studies have shown that an improved type of vacuum pre-cooling could effectively restrain the respiration rate and POD activity of Chinese flowering cabbage, pak choi, and cabbage, helping to maintain relatively high vitamin C and chlorophyll content and preserve crop appearance [39]. However, ethylene treatment can accelerate the deterioration in the quality of Chinese flowering cabbage [28]. Therefore, it was speculated that 0.5 ppm thyme essential oil treatment might inhibit ethylene signal transduction from controlling the respiratory intensity of Chinese flowering cabbage during storage, avoid the excessive loss of nutrients, and finally preserve the nutritional quality of cabbage.

### 3.3. Thyme Essential Oil Treatment at 0.5 ppm Can Improve the Storage Properties of Chinese Flowering Cabbage by Regulating the Activities of POD and CAT Enzymes

**Figure 10:**
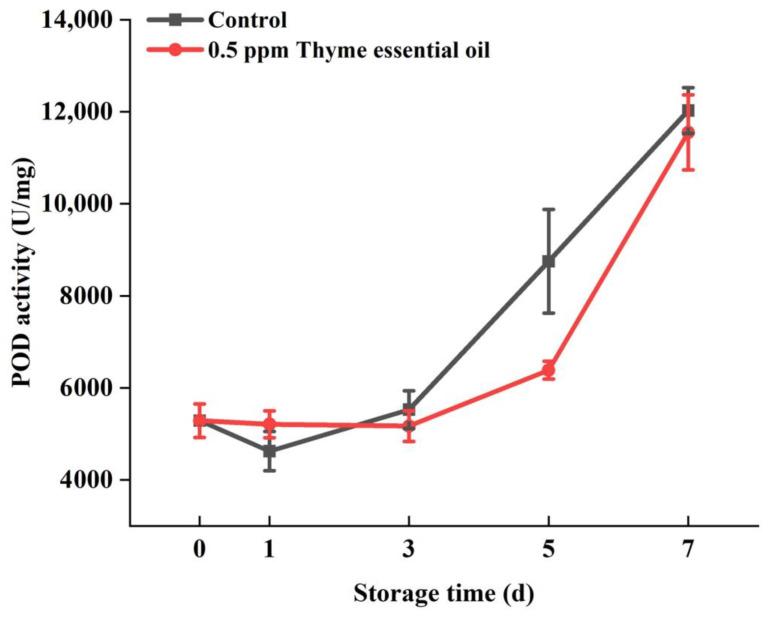
Effect of 0.5 ppm thyme essential oil treatment on the POD activity of Chinese flowering cabbage during storage for 7 days.

POD plays an important role in the process of plant growth, development, and stress resistance. It can clear hydrogen peroxide in vivo by catalyzing the oxidation of phenolics, thereby protecting cells from oxidative damage. In this experiment, it was found that the POD activity of Chinese flowering cabbage increased rapidly after storage for 3 days, while the POD activity of the 0.5 ppm thyme essential oil treatment group remained relatively low during the early storage period (0–5 days). Interestingly, the activity of POD increased quickly again after storage for 5 days (Figure 10). Therefore, it was speculated that 0.5 ppm thyme essential oil treatment can delay the enhancement in POD enzyme activity. Moreover, CAT is another critical antioxidant enzyme in plants that can also decompose excessive hydrogen peroxide in cells and reduce its poisoning effect. Along with the process of ripening and senescence in fruits and vegetables, active oxygen and other harmful substances accumulate continuously in vivo, which, to some extent, also promotes the increase in antioxidant enzyme activity. It was confirmed in this study that CAT activity in the control group was steadily reinforced with storage time, while the 0.5 ppm thyme essential oil treatment group maintained a relatively low enzyme activity for the first 5 days, followed by a prompt augmentation on the seventh day, reaching a level that was 14.25% higher compared with the control, and displayed a similar variation trend as that seen in POD (Figure 10). Accordingly, it is presumed that 0.5 ppm thyme essential oil treatment can promote the enhancement in CAT enzyme activity to remove hydrogen peroxide.

**Figure 11:**
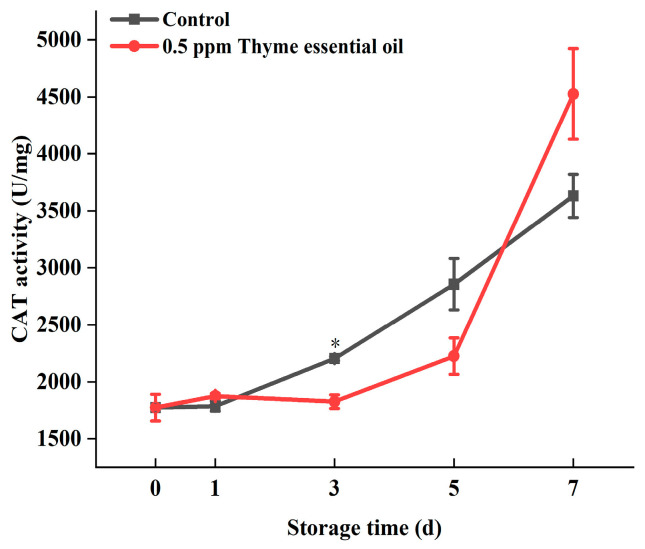
Effect of 0.5 ppm thyme essential oil treatment on CAT activity in Chinese flowering cabbage during storage for 7 days. Each value represents the means ± SE of three replicates. Asterisks indicate significant differences (* *p* < 0.05) between the 0.5 ppm thyme essential oil treatment group and the control at each time point during storage.

ROS in a certain concentration range, such as hydrogen peroxide and superoxide free radicals, can improve plant disease resistance and promote the activity of antioxidant enzymes. However, the continuous accumulation of ROS causes oxidative damage to cells and accelerates the senescence of fruits and vegetables. Therefore, fruit and vegetable tissues must maintain a balance between ROS accumulation and scavenging. Studies have shown that plant essential oils can enhance the activities of SOD, POD, CAT, and APX during postharvest storage of carambola, raspberry, and other fruits, increasing their antioxidant activity [40,41]. Among them, POD, as an antioxidant enzyme, can degrade hydrogen peroxide and is also a vital enzyme in the process of lignin synthesis in plants. However, stemming from the increased activity of POD, lignification is unfavorable for the taste of fruits and vegetables. Our previous studies revealed that lignin biosynthesis could be regulated by hydrogen peroxide signaling [42], and lignin deposition around the vascular tissue was involved in the senescence of Chinese flowering cabbage after harvest [43]. Xie et al. proved that 1-methylcyclopropene treatment could suppress enhanced POD activity in fresh common beans and reduce the degree of lignification [44]. Li et al. similarly found that melatonin treatment could delay the increase in POD activity in bamboo shoots and lessen the extent of lignification [45]. Moreover, 0.5 ppm thyme essential oil treatment delayed the enhancement in POD activity during the storage of Chinese flowering cabbage (Figure 10), which indicates that POD also participates in the synthesis of lignin during the storage of Chinese flowering cabbage. Furthermore, a low concentration of hydrogen peroxide treatment could delay leaf senescence in Chinese flowering cabbage during storage, alleviating oxidative damage by activating the antioxidant defense system [46]. An increase in hydrogen peroxide content in plant cells could induce an augmentation in CAT gene expression and activity [47]. Similarly, the activity of CAT in Chinese flowering cabbage increased continuously during storage in this study, which is consistent with the deterioration in its appearance and quality. In addition, 0.5 ppm thyme essential oil treatment increased the CAT activity of Chinese flowering cabbage during anaphase storage (Figure 11). Interestingly, Hasan et al. [48] previously found that chitosan coating applications loaded with thyme volatile oil could considerably promote the activities of CAT and SOD enzymes, which are associated with shelf-life extension for sweet basil leaves. In this study, it was found that the activities of POD and CAT in Chinese flowering cabbage increased gradually with enhanced ROS content during the storage process, while 0.5 ppm thyme essential oil treatment delayed the increment in POD activity, reduced the degree of lignification, improved CAT enzyme activity, and avoided excessive oxidative damage in the later storage period, ultimately improving the storage quality of Chinese flowering cabbage.

## 4. Conclusions

Herein, it was revealed that 0.5 ppm thyme essential oil treatment can maintain the appearance and quality of Chinese flowering cabbage during storage at 15 °C and 90% RH, delay the degradation of chlorophyll and inhibit the rise in the relative conductivity in leaves. Moreover, the nutritional quality of Chinese flowering cabbage can also be sustained via treatment during the preservation period. This study also found a delay in the reduction in vitamin C, soluble solids, soluble sugar, and soluble protein. Furthermore, 0.5 ppm thyme essential oil treatment delayed the lignification of Chinese flowering cabbage by inhibiting the increase in POD activity and promoting CAT activity in the later storage period, cleaning up hydrogen peroxide in vivo and thus improving its storage properties. However, the molecular mechanisms of essential oil-related genes involved in chlorophyll degradation and antioxidation during the storage of Chinese flowering cabbage, which might be regulated by thyme essential oil treatment, need to be further elucidated. In summary, 0.5 ppm thyme essential oil treatment can effectively postpone the senescence process of Chinese flowering cabbage during storage. Therefore, thyme essential oil, due to its antibacterial and antioxidant properties, represents a safe and efficient green preservative with great commercial application potential.

## Data Availability

The original contributions presented in the study are included in the article/Supplementary Material, further inquiries can be directed to the corresponding author.

## Supplementary Materials

The following supporting information can be downloaded at: https://www.mdpi.com/article/10.3390/foods14213704/s1, Figure S1: Effect of plant essential oil treatments on the appearance of Chinese flowering cabbage after storage at 15 °C for 7 days, Figure S2: Effect of different concentrations of thyme essential oil treatments on the appearance of Chinese flowering cabbage after storage at ambient temperature for 7 days.
