# Supplementary material for: Treatment with Thyme Essential Oil Delays Loss Reductions in Postharvest Chinese Flowering Cabbage
Source: Foods. 2025 Oct 29;14(21):3704. doi: 10.3390/foods14213704 (PMC12609979; doi:10.3390/foods14213704)
Supplement: Supplementary file 1 [file foods-14-03704-s001.zip › foods-3936105-supplementary.pdf]

Supplementary material

Figure S1: Effect of plant essential oil treatments on the appearance of Chinese flowering cabbage after storage at 15℃ for 7 days.

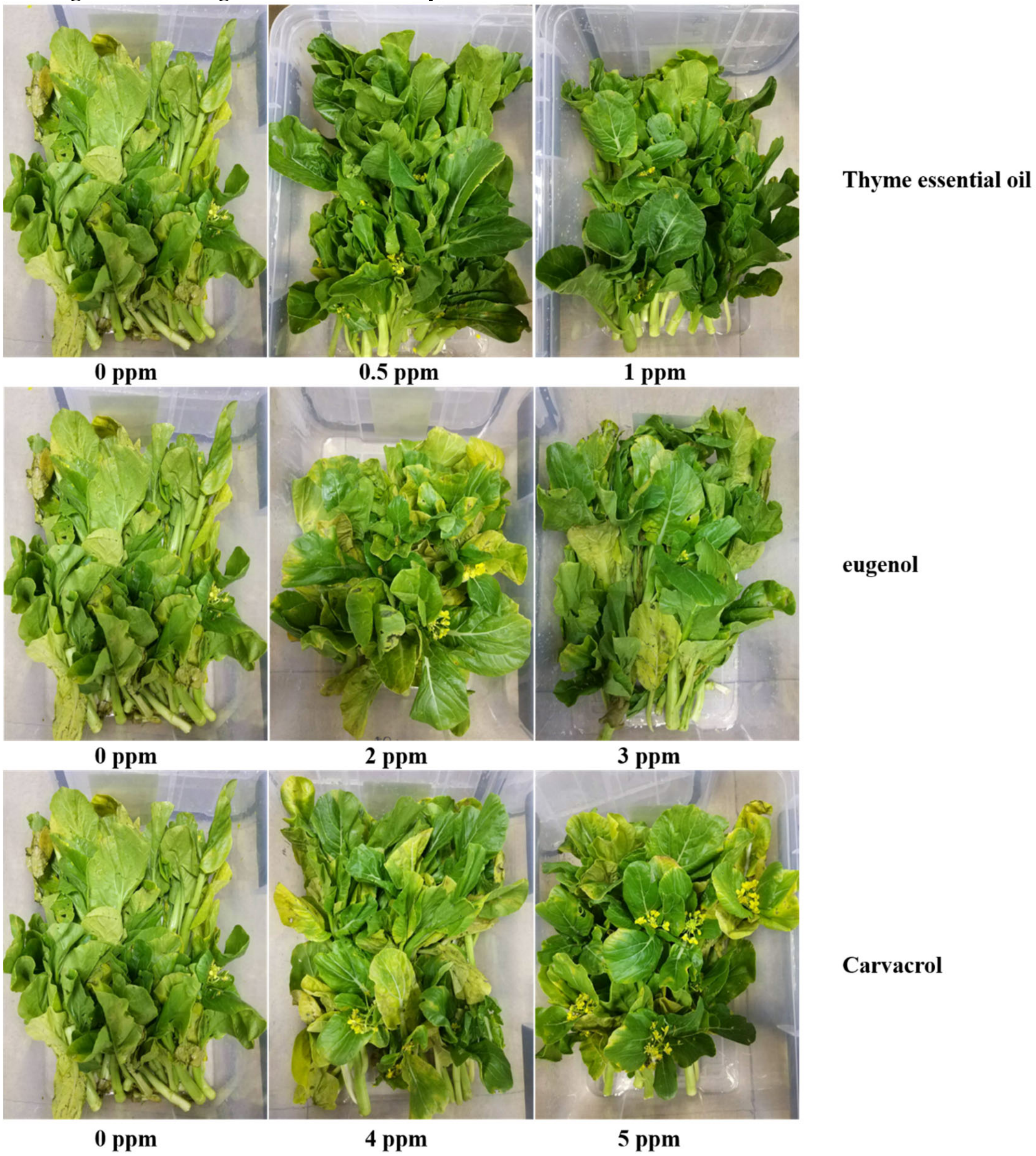

Figure S2: Effect of different concentrations of thyme essential oil treatments on the appearance of Chinese flowering cabbage after storage at ambient temperature for 7 days

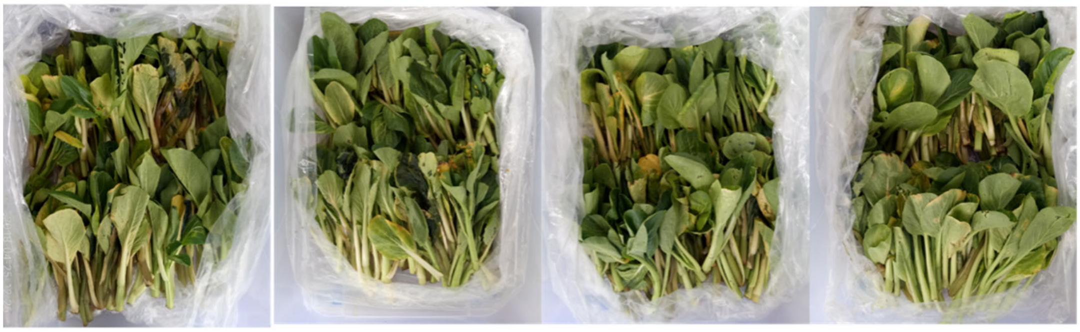

Control | 1 ppm thyme essential oil | 2 ppm thyme essential oil | 4 ppm thyme essential oil
